# A Practical Treatise on the Diseases of the Testis, and of the Spermatic Cord and Scrotum, with Illustrations

**Published:** 1844-07

**Authors:** 


Art. VII.—*A Practical Treatise on the Diseases of the Testis, and of the Spermatic cord and Scrotum. With illustrations.* By T. B. Curling, Lecturer on Surgery and Assistant Surgeon to the London Hospital, Surgeon to the Jews' Hospital, &c. Edited by P. B. Goddard, M.D., M.A.P.S., M.A.N.S., Demonstrator of Anatomy in the University of Pennsylvania, &c. Philadelphia: Carey & Hart. 1843. 8vo. pp. 566.

The diseases of which this volume treats awaken in patients, it is well known, a degree of solicitude greater than is excited by maladies of a more fatal character, at the same time that they are often the most embarrassing that are presented to the medical man. A complete monograph on the subject has, therefore, been much needed by the profession in our country. That is now afforded in the work the title of which is given at the head of this article. Mr. Curling is admitted, on all hands, to have produced a treatise of uncommon excellence, in which is comprised the best extant information relative to affections of the testis. The volume is one of great neatness. The paper is of excellent quality, the type fair, and the wood-cuts finely executed. Altogether it is a work which one feels safe in recommending in the strongest terms to the profession.

The author begins with a concise account of the anatomy of the organs embraced in his treatise, which will be found by the student an interesting introduction to the diseases of the parts. Concerning the functions of the testis he has the following remarks:

"A boy reared in the country of a moral disposition and active habits does not experience the force of sexual appetite till the body is well developed, and nature permits the gratification of his passion without injury to the constitution. It is otherwise, however, with the young inhabitant of a city and the associate of the dissolute. Before puberty is complete and the body fully formed, desire is prematurely aroused by impure addresses to the imagination, and the development of the testis is hastened, and they are called into action earlier than if they were left solely to the impulse of nature.

Unlike the animal creation the testes in men are ready at all seasons to perform their office. The desires subside and the secretion of the semen becomes languid as life advances, though they seldom cease entirely till the age of sixty-five or seventy. Indeed, I have several times discovered spermatozoa in the testes of men upwards of seventy years of age, and once in the testicle of a tailor who died at the age of eighty-seven; and there are instances on record of persons retaining the procreative faculty to the age of one hundred years; but in these cases, as in the well-known instance of old Parr, the general bodily powers were also preserved in a very extraordinary degree."

But we shall not stop to make quotations from this part of the work, although it contains much matter which may be studied with advantage. The views of the author are marked by correct judgment, and a sound tone of morality.

Part II is devoted to a consideration of the diseases of the testis, of which the first chapter treats of congenital imperfections and malformations. We pass over this chapter, as possessing less practical interest than many other portions of the work.

The matter of the second chapter, on atrophy of the testis, is important. The causes of atrophy are given in detail, some of which are shown to be under the control of the patient, or of his medical attendant. Among these may be cited an impeded circulation, pressure, want of exercise, and inflammation. Atrophy has resulted from the pressure of a truss awkardly applied, and from that of a hydrocele or a hæmatocele, and is more frequently a consequence of inflammation. It is one of the evils to be apprehended from an attack of mumps. Abuse of the organs is another cause of atrophy, of which many instances are given. The long-continued use of iodine has been supposed to induce this condition of things, but our author calls the correctness of this belief in question. Most of the causes of atrophy lie too deep to be reached by the surgeon. Once established, from whatever cause resulting, we have but little power by any mode of treatment to arrest the decay of the organ. The treatment consists in avoiding the causes.

Injuries of the testis are treated of in the third chapter, of which self-castration makes one, and of which interesting instances are related by our author, who remarks that such cases usually do well.

Much space is devoted to the subject of *hydrocele*, and is given with a minuteness which will satisfy the most curious inquirer. We make a few extracts from this chapter, and first as to the termination of the disease:

"A hydrocele may disappear without any treatment whatever. In infants this is a constant occurrence, but in adults is extremely rare. Mr. Pott has recorded two instances of the spontaneous disappearance of a confirmed hydrocele in the adult. One is the case of a gentleman forty-five years of age, in which the dropsical collection dispersed during six weeks' confinement for a severe fit of gout. The other is the case of a middle-aged man, who whilst intoxicated fell down and struck his scrotum against a piece of scaffolding, which caused considerable ecchymosis. This disappeared in about a fortnight, when it was observed that the hydrocele was much less in size than it was before the accident. In about three weeks more the whole of it had subsided, and it did not afterwards return. Sir B. C. Brodie also mentions that he has met with two examples of the spontaneous cure of hydrocele. In one of them the removal of the disease appeared to have resulted from inflammation set up in the sac.* A hydrocele has also been known to disappear permanently after an attack of orchitis, consequent upon the extension of inflammation from the urethra. But these cases are exceptions to the general rule, and are not to be taken into account in determining upon the treatment to be adopted."

In the fourth volume of this journal we related the case of an aged gentleman who was cured of hydrocele by a fall from his horse. The inflammation of the sac resulting from the vi-

---

* London Med. Gazette, vol. xiii., p. 90.

olence done to it in the fall, or more probably the rupture of the tunic, led to the absorption of the fluid. It is now more than three years since the subsidence of the tumor took place; but we were informed by our old friend, the subject of the complaint, a short time since, that the water was again collecting. This gentleman is now nearly 80 years of age, and does not think of submitting to an operation.

Our author does not highly esteem external remedies in cases of the disease affecting adult subjects. Such treatment is annoying, and only leads to loss of time. Occasionally the distended tunica vaginalis is ruptured by accidental violence, and the fluid finds its way into the scrotum. In some cases the hydrocele is thus permanently removed, but in general the fluid collects again. An operation is the only thing that can be depended upon as a cure. It may be of a *palliative* or of a *radical* character. The palliative operation consists in puncturing the tumor, which may be done with a lancet or trocar, so as to allow the escape of the fluid contained in the tunica vaginalis; but the relief it affords is only temporary, and the operation is again demanded after some time.

"I have had patients who for many years have been satisfied with the relief afforded by an annual operation; and in one case the fluid did not collect in a sufficient quantity to need removal for four years, when I drew off no more than sixteen ounces. In other instances patients have returned to have the fluid evacuated again at the expiration of two or three months, and even of a much shorter period. Indeed, I have known the hydrocele to regain its former size in the course of two or three days. Many persons suffer pain and uneasiness from only a small quantity of fluid, whilst others experience but little inconvenience until the hydrocele has attained a large size. In most cases the patient's feelings will be the best guide in indicating the necessity for a repetition of the operation."

Another mode of removing the fluid is by a puncture with a needle. The water instead of escaping externally, as in the former operation, gradually infiltrates the cellular tissue surrounding the sac, whence it is afterwards removed by absorption.

"Mr. Lewis, surgeon, of London, is entitled to the credit of having first recommended acupuncture to his professional brethren on the grounds of practical experience of its efficacy;* though no doubt can be entertained that the plan had been previously resorted to by other surgeons, who had regarded it as either too simple or too unimportant to deserve a formal notice, or who perhaps did not sufficiently appreciate its value.† Mr. Lewis' practice is to puncture the tumor with a fine needle until a drop of fluid oozes out on withdrawing it, and in a few days the hydrocele will entirely disappear. The absence of danger, the slow re-accumulation of the fluid, and the simplicity of the operation, are the advantages which he considers to be obtained by this mode over the operation of removing the fluid at once."

The operation for a radical cure is different in the hands of different surgeons. All the plans are described in this work—incision of the sac; caustic applied to the integuments; a tent introduced into the tunica vaginalis; a seton passed through the sac; and injection of the sac with a stimulating fluid. Mr. Curling prefers the latter mode, which is attended, according to his experience, by results much less severe than any of the other methods. We shall not follow him through the various details of the operation, for which the surgeon will refer to works on the subject; but the importance of the following remarks justifies their citation:

"I never inject a recent hydrocele, or one, however small, the first time of tapping, as it occasionally happens that the fluid ceases to collect after its evacuation. When the fluid amounts to more than ten or twelve ounces the hydrocele is unfit for injection, because the extent of the serous surface in large hydroceles is liable to render the effects of this treatment more severe than is desirable. In these cases it is better to draw off the fluid, and then wait until a

---

* Lancet, vol. ii. 1835–'36, p. 209.

† Vide note from Mr. Lewis on the Treatment of Hydrocele; Med. Gaz., vol. xix., p. 789.

smaller quantity is formed, when the operation may be undertaken with less risk. The surgeon should also be careful to ascertain that the dropsical effusion is not dependent on existing disease of the testis. A man was admitted into the London Hospital with a double hydrocele on purpose to undergo the operation for a radical cure. He had been suffering for some time previously from disease of the larynx, which increased soon after his admission, and caused suffocation and death. On examination of the testes, deposits of concrete pus were found in the substance of both the glands. In this case, had his state of health permitted of an operation, after removal of the fluid the diseased condition of the testes would probably have been detected, and injection, which could only have operated injuriously, would have been abandoned. The fluid effused around the diseased testis by producing pressure sometimes causes pain, and it may then be evacuated with benefit; but I need scarcely add that to attempt the permanent removal of the hydrocele whilst the original disease remains unsubdued, would be both fruitless and hurtful. The disease producing the enlargement of the gland must be treated without reference to the effusion, and it will commonly be found that as the affection of the testis subsides the hydrocele likewise disappears. Thus in several cases of hydrosarcocele consequent on an attack of orchitis, in which after drawing off the fluid the testis has been found tender as well as enlarged, I have succeeded by the exhibition of small doses of mercury, and by strapping, or by applying mercurial or iodine ointment to the part, in subduing the chronic inflammation of the gland and effecting the cure of the hydrocele. In some instances, however, inflammation of the testis or epididymis is the primary disease, the hydrocele and disposition to an increased secretion remain long after all morbid action has ceased. The case must then be regarded in the same light, and treated in the same way, as ordinary hydrocele; but the surgeon will do right to recollect that where inflammation has once been excited, its return is usually induced more readily and by slighter stimulating causes than before."

*Electro-puncture* is a modification which has been proposed in the operations for hydrocele. The American editor of this work cites a case cured by this method, in which the seton and injections had both failed. The operation is performed by introducing two acupuncture needles into the sac, and connecting one to the positive and the other to the negative pole of a galvanic battery.

Of the complicated forms of hydrocele we will attempt no account, since it would be impossible in our prescribed limits to give even a satisfactory outline of the various modifications of the complaint, without omitting entirely a notice of other interesting topics discussed in this work. In like manner we shall pass over the chapter on hæmatocele of the testis and of the spermatic cord, to make a few extracts from the one which treats of orchitis.

"Inflammation of the testis," remarks our author, "is far more frequently a consecutive affection than a primary, the inflammation being transmitted from the urethra by the course of the vas deferens." It is then known by the term *hernia humoralis.* The causes are, mechanical injuries, mumps, 'great excitement of the organs without the opportunity of indulging the passions,' irritation of the urethra by foreign bodies, and the inflammation of that membrane in gonorrhœa. Of all these gonorrhœa is by far the most common cause, and as often gives rise to it when copaiba and cubebs are not used as when they are. The disease seems occasionally to partake of a rheumatic character, and to be induced by cold. Changes of a serious nature in the testis not unfrequently result from orchitis, the organ is more liable to inflammation afterwards, and often remains more sensitive, becoming swollen and painful on slight causes. Having given the general treatment in inflammation of this gland, our author proceeds:

"The cure of acute orchitis has been much facilitated, of late years, by the application of a mode of treatment which has been found of great service in relieving certain forms of acute inflammation in other parts of the body, viz. *compression.* The object of compression is to afford support to the weakened vessels; and in acute inflammation of the integuments, when properly applied for this purpose, and not so firmly as to produce pressure and arrest the circulation, it often proves a very valuable method of treatment. Dr. Fricke, of Hamburg, I believe, first suggested the practice of treating both acute and chronic orchitis by compression, applied to the testis by means of adhesive plaster. In an early report of this practice, he states that out of fifty-one cases of acute orchitis eighteen were treated by the ordinary means, and thirty-three by compression. In the latter cases the average duration of the disease was nine days, whilst in the former it was thirteen. In cases treated more recently, after improvements had been made in the mode of applying the compression, the result was still more favorable. This practice has since been adopted in Paris by Cullerier and Ricord; and in this country by Mr. Langston Parker, Mr. Acton, my colleague Mr. Hamilton, myself, and others, and, I am informed, by the army surgeons generally. Some care is required in making the application, which I perform as follows: The patient being placed in the recumbent position, with the testis raised, is to remain there three or four minutes, in order to allow the vessels of the gland to become as empty as possible. The parts are to be shaved; and some *emplastrum plumbi* must be cut into strips, about three-quarters of an inch in width, and eight or nine inches in length. The opposite testis and side of the scrotum being drawn away from the diseased one, so as to render the integuments of the latter quite tense, the first strap is to be placed circularly round the cord, just above the testis, as tightly as the patient can bear it. The second strap is to be placed in an opposite direction, from behind forwards, at the side of the testis near the septum. The third strap is to be applied below the first, so as partly to overlap it; and the fourth in like manner, internal to the second; and so on in succession, until the straps meet, and the whole of the testis is covered, and evenly compressed. A few additional straps may afterwards be applied where most needed to afford support, and keep the others in place; the parts are afterwards to be supported in a suspensory bandage. The strapping generally requires to be re-applied in the course of twenty-four hours. When the patient rises after its application he feels completely relieved from the aching pain and sense of weight; and patients who have remained in bed in consequence of the pain have immediately been able to get up and walk about. Some surgeons recommend the application of compression at the onset of the inflammatory attack; and if the inflammation be moderate this may be done with advantage, so that when concealment is desirable the patient may be able to continue his usual avocations. But in decidedly acute orchitis it is better to commence with an active purge, the tartar emetic, and, if necessary, depletion, and to enjoin rest; and then, after twenty-four or forty-eight hours, to resort to compression: for in active inflammation of the testis compression is not always sufficient without other antiphlogistic means, and if solely depended on, may disappoint expectations. But after the more acute symptoms are relieved compression greatly facilitates the cure, affording relief from pain, causing the rapid subsidence of swelling, and removal of all effused matter. In cases of a chronic character, or to remove the thickening and induration of the epididymis and cord so commonly observed after acute consecutive orchitis, the testis may be strapped with the *emplastrum ammoniac cum hydrargyro;* or iodine or mercurial ointment may be first applied to the scrotum, and then the strapping, so as to combine the advantages of these applications and of compression. The removal of the chronic enlargement and induration may be further promoted by the exhibition of small doses of mercury. In these cases I sometimes keep up counter-irritation by painting the scrotum on the side with the following solution—*Iodin.* ʒj., *Potass. Iodid.* ʒss., *Sp. Vin. Rect.* ℥j.; repeating the application every third or fourth day, until the gland is restored to the healthy state. Blisters are sometimes used: but they are too irritating to be applied to the scrotum, and are even liable to cause mortification."

Tubercular disease of the testis, carcinoma, encephaloid, and various other degenerations of the gland are described in subsequent chapters, of which we shall not pause to make a single remark, as there still remain to be noticed other topics of greater interest to the practical surgeon. One of these is irritable testis, of which our author gives the following graphic description:

"A patient suffering from an irritable testis cannot in many cases bear the least pressure on the gland, not even the contact of his dress: he shrinks when the part is handled in the most gentle manner; and the motions of the testis often occasion so much uneasiness that he is prevented from taking exercise, and is compelled to remain constantly at rest in the recumbent position. There is no perceptible alteration in the parts, except occasionally a degree of fulness, more particularly in the spermatic cord; slight varicose dilatation of the veins, and a relaxed state of the scrotum. The complaint is usually tedious, and lasts many months. The persons subject to it are those of a weak and irritable habit, who are dyspeptic or hypochondriacal, and unequal to much bodily or mental exertion. In this affection all enjoyment of life and its pleasures disappears; the sufferers concentrate their thoughts upon their maladies; they fancy they shall never get cured; and whilst some become uneasy as to the effect of the complaint in impairing the integrity of the gland, and rendering them impotent, others as urgently desire castration as the sole means of relief from their distress."

In the treatment of this affection the first point is to remove the cause, which not unfrequently is connected with abuse of the sexual organs, after which we are to aim at improving the general tone of the system by tonics, of which the preparations of iron are the best. Cold bathing and sponging are also beneficial, and relief is often afforded by a change of scene and air which diverts the patient's mind from his complaints. In cases where it depends on an irritable condition of the prostatic part of the urethra, accompanied by involuntary seminal emissions, the author advises the application of nitrate of silver to the seat of the irritation.

The chapters on neuralgia of the testicle, and sympathetic and fundamental disorders of the organ are full of most interesting details, but they are of such a character that it would be impossible to condense them. With a single extract more, which is taken from the chapter on varicocele, we shall bring our imperfect notice of this compendious and most excellent treatise to a close. We have not pretended to exhibit its principles and practical instructions in outline, much less to give such a summary of its matter as would suffice for the practitioner. But we have exhibited its merits at sufficient length to satisfy our readers, that it is a work to which they may refer with profit concerning the diseases of the important organ of which it treats.

"The indication for the perfect cure of varicocele is not merely to aid and support the dilated and weakened vessels, but so far to relieve them of the superincumbent weight of the blood as to enable them to return to their natural dimensions, and recover their tone so as duly to carry on the circulation. The first indication may be fulfilled to a certain extent by the suspensory bandage, and in some cases permanently by the excision of a portion of the scrotum; and in mild cases these plans are sufficient to give all the relief required, and to prevent the extension of the disease: the latter proceeding may be resorted to whenever the patient is tired of wearing the bandage, and is willing to submit to an operation which, though painful, is not under ordinary circumstances attended with danger. But neither artificial support, nor excision of the scrotum, is capable of fulfilling the second indication—of reducing the size and thickened coats of the dilated veins. The only plan which appears to be fully adapted to effect this object is firm, steady, and continued pressure on the spermatic veins at the ring by a well-adjusted truss. At present our experience of this mode of treatment is too limited to admit of any opinion of its efficacy being confidently expressed; but I look with no slight interest to the result of further trials of a remedy which seems to me to be based on no sound views of the pathology of the disease. This plan appears to be particularly applicable to cases of varicocele in young persons, whose reparative powers would be sufficient to restore the veins when relieved of pressure to a healthy state." Y.

